# Sprains and Allied Injuries of Joints

**Published:** 1910-03

**Authors:** 


**Sprains and Allied Injuries of Joints.** By R. H. ANGLIN WHITELOCKE, M.D., M.C. Pp. xvi, 241. London: Oxford Medical Publications. 1909.

We welcome this book as a clinical treatise based upon the exceptional experience of the writer in this branch of surgery. It may be regarded as a counterpart, from a different point of view, of the excellent clinical work of Sir Wm. Bennett upon the same subject. The illustrations are particularly valuable, being photographs of cases, skiagrams, and specimens; and they are exceptional in providing examples of the ill-effects of treatment by bone-setters, such as we have not seen before, and of some of the rarer lesions of bones accompanying so-called sprains. It is perhaps strange, however, that no illustration is given of fracture of the tuberosity of the fifth metatarsal bone, of which we have seen several examples, and which is more frequent than recognised. There are excellent photographs showing the technique of operation for exploration of the knee-joint; and we regard the book as a useful contribution to surgical literature, especially when read in conjunction with other works of its class.

